# Impact of Type 2 Diabetes on Impaired Kidney Function in Sub-Saharan African Populations

**DOI:** 10.3389/fendo.2016.00050

**Published:** 2016-05-30

**Authors:** Sally N. Adebamowo, Adebowale A. Adeyemo, Fasil Tekola-Ayele, Ayo P. Doumatey, Amy R. Bentley, Guanjie Chen, Jie Zhou, Daniel Shriner, Olufemi Adetola Fasanmade, Godfrey Okafor, Benjamin Eghan, Kofi Agyenim-Boateng, Jokotade Adeleye, Williams Balogun, Albert G. Amoah, Samuel Owusu, Joseph Acheampong, Thomas Johnson, Johnnie Oli, Clement A. Adebamowo, Charles N. Rotimi

**Affiliations:** ^1^Center for Research on Genomics and Global Health, National Human Genome Research Institute, National Institutes of Health, Bethesda, MD, USA; ^2^University of Lagos, Lagos, Nigeria; ^3^University of Nigeria Teaching Hospital, Enugu, Nigeria; ^4^Kwame Nkrumah University of Science and Technology, Kumasi, Ghana; ^5^University of Ibadan, Ibadan, Nigeria; ^6^University of Ghana Medical School, Accra, Ghana; ^7^Department of Public Health and Epidemiology, Greenbaum Cancer Center, Institute of Human Virology, University of Maryland School of Medicine, Baltimore, MD, USA

**Keywords:** impaired kidney function, type 2 diabetes, kidney disease, sub-Saharan Africa, diabetic kidney disease

## Abstract

**Background:**

Diabetes is a leading risk factor for impaired kidney function, an indicator of chronic kidney disease. The aim of this study was to examine the association between type 2 diabetes (T2D) and impaired kidney function among adults in sub-Saharan Africa (SSA).

**Methods:**

Participants were enrolled from Ghana, Kenya, and Nigeria. Impaired kidney function was based on an estimated glomerular filtration rate <60 ml/min/1.73 m^2^. Using logistic regression models, we conducted case–control analyses to estimate the multivariate-adjusted association of T2D and kidney function.

**Results:**

We used data from 4815 participants for whom the mean (SD) age was 48 (15) years, 41% were male and 46% had T2D. Those with T2D were more likely to have impaired kidney function [13.4% (95% CI: 11.9–14.7)] compared to those without T2D [4.8% (95% CI: 4.0–5.6)], *p-*value <0.001. The multivariate odds ratio of impaired kidney function among those with type 2 diabetes was 1.50 (95% CI: 1.17–1.91) *p*-value = 0.001, compared to those without T2D. Also, individuals with T2D who were at least 60 years old, obese, hypertensive or dyslipidemic were more likely to have impaired kidney function compared to those without T2D.

**Conclusion:**

T2D was associated with 50% increased risk of impaired kidney function in this sample of adults from SSA. Interventions targeted at prevention, early diagnosis, and management of T2D are likely to reduce the burden of kidney disease in SSA.

## Introduction

Non-communicable diseases (NCD), such as diabetes and kidney disease, have replaced communicable diseases as the most common causes of premature death worldwide. An estimated 80% of the NCD burden occurs in low- or middle-income countries (1). Diabetes is an important risk factor for kidney disease; about a third to half of the individuals with diabetes develop kidney disease (2, 3). Globally, while the age-standardized rates for chronic kidney disease (CKD) due to hypertension decreased by 22.4%, the rates for CKD due to diabetes mellitus increased by 10.6%, between 1990 and 2013 (4). In all developed and many developing countries, diabetes and hypertension are the leading causes of CKD (5). Given the projection that the number of people with diabetes in sub-Saharan Africa (SSA) will increase by 141% from 14.2 million in 2015 to 34.2 million in 2040 (6), the potential for an increasing burden of kidney disease in SSA is high.

There is a dearth of data on the risk of impaired kidney function among individuals with type 2 diabetes (T2D) in SSA. The studies from SSA that have reported on the prevalence of kidney disease often had characteristics that limit their interpretation, including lack of standard definitions of kidney disease and small sample size of typically <1000 adults (7–18). The objective of this study was to examine the association between T2D and impaired kidney function, an indicator of CKD, in a large sample of about 5000 sub-Saharan Africans and to examine the variation of the association between T2D and risk of impaired kidney function by age, hypertension, body mass index (BMI), and dyslipidemia.

## Materials and Methods

### Study Population

The study participants were adults who were at least 18 years old. Individuals with T2D were enrolled from university medical centers in Ibadan, Enugu, and Lagos in Nigeria, Accra, and Kumasi in Ghana and Eldoret in Kenya as part of a genetic epidemiology study of T2D in sub-Saharan Africans. Individuals without T2D were enrolled from surrounding communities of the respective university medical centers. At each site, research nurses approached potential participants at outpatient clinics and community centers, discussed the study objectives, and obtained informed consent from willing participants. About 96% of those who were approached agreed to participate in the study. After providing informed consent, all participants provided information on demography, social, and lifestyle factors, and underwent a clinical examination that included a medical history, clinical anthropometry, blood pressure measurements, and blood sampling for measurement of cardiometabolic parameters. Weight was measured in light clothes on an electronic scale to the nearest 0.1 kg, and height was measured with a stadiometer to the nearest 0.1 cm. BMI was computed as weight in kilograms divided by the square of the height in meters. Ethical approval for this study was granted by the Institutional Review Board at each study site, Howard University and the National Institutes of Health.

### Ascertainment of Type 2 Diabetes

The definition of T2D was based on the following criteria: a fasting plasma glucose concentration ≥126 mg/dl (7.0 mmol/l) or a 2-h post-load value in the oral glucose tolerance test ≥200 mg/dl (11.1 mmol/l) on more than one occasion. Alternatively, a diagnosis of T2D was accepted if an individual was on pharmacological treatment for T2D and review of clinical records indicated adequate justification for that therapy. The detection of autoantibodies to glutamic acid decarboxylase and/or a fasting C-peptide ≤0.03 nmol/l was used to exclude probable cases of type 1 diabetes. Participants without T2D were those who had FPG <100 mg/dl (5.6 mmol/l) or 2-h post-load of <140 mg/dl (7.8 mmol/l) and no symptoms suggestive of diabetes (the classical symptoms being polyuria, polydipsia, and unexplained weight loss).

### Ascertainment of Kidney Function

Serum creatinine levels were determined using a Jaffe reaction without deproteinization on a COBAS Integra 400 analyzer (Roche Diagnostics, IN, USA). Currently, glomerular filtration rate (GFR) is considered to be the best indicator of renal function (19). Given that results from studies evaluating the performance of estimated GFR (eGFR) equations in black Africans have been inconsistent (20, 21), we calculated race and gender-specific eGFR using the Modification of Diet in Renal Disease (MDRD) and Chronic Kidney Disease Epidemiology Collaboration (CKD-EPI) equations. A recent evaluation of eGFR equations among Africans with T2D showed that the MDRD was associated with lower bias compared to the CKD-EPI and Cockcroft–Gault equations (22). Thus, we used eGFR calculated from the MDRD equation for our multivariate analyses. We defined impaired kidney function as a dichotomous trait based on an eGFR <60 ml/min/1.73 m^2^, which corresponds to CKD stage 3 or worse based on the new Kidney Disease Improving Global Outcomes (KDIGO) staging recommendations (23), when accompanied by signs of kidney damage. Cases were defined as individuals with impaired kidney function (eGFR < 60 ml/min/1.73 m^2^) and controls as individuals without impaired kidney function (eGFR ≥ 60 ml/min/1.73 m^2^).

### Statistical Analyses

Means and proportions of the participants’ baseline characteristics were calculated. Tests of significance to compare means and proportions between participants with diabetes and those without diabetes were conducted using *t*-tests, Fisher’s exact, and Mantel–Haenszel tests. To examine the association between impaired kidney function (eGFR < 60 ml/min/1.73 m^2^) and T2D, logistic regression models were used to estimate odds ratios (OR) and 95% confidence intervals (CI). Multivariate models were adjusted for age, sex, BMI, hypertension, high-density lipoprotein cholesterol (HDLc), low-density lipoprotein cholesterol (LDLc), and triglycerides. Hosmer and Lemeshow goodness-of-fit test was used to examine whether the model was a good fit. To examine potential effect modification of the association between T2D and risk of impaired kidney function, multivariate models were stratified on age (<60 vs. ≥60 years), sex (male vs. female), BMI [normal (18.5–24.9 kg/m^2^), overweight (25.0–29.9 kg/m^2^), obese (≥30 kg/m^2^)], hypertension (yes vs. no), HDLc (<1.04 vs. ≥1.04 mmol/l), LDLc (<4.14 vs. ≥4.14 mmol/l), and triglycerides (<2.26 vs. 2.26 mmol/l). All *p* values are two-sided, and the analyses were conducted using SAS 9.3 for Unix (SAS Institute Inc., Cary, NC, USA).

## Results

The baseline characteristics of the study participants are presented in Table 1. A total of 4815 participants (421 cases and 4394 controls) were included in the analyses. The mean age (SD) was 48 (15) years, and 41% (1984/4815) were male. Participants with impaired kidney function were more likely to be older, female, hypertensive, and diabetic, and have higher levels of LDL and triglycerides (Table 1).

**Table 1:** **Characteristics of the study participants by case–control status**.

	Impaired kidney function, eGFR (MDRD)		*p*-value
	Cases (<60 ml/min/1.73 m^2^)	Controls (≥60 ml/min/1.73 m^2^)	
*n*	421	4394	
Age, years	58.7 ± 10.4	47 ± 14	<0.01
Male sex, %	37	63	0.08
Body mass index, kg/m^2^	26.6 ± 4.8	26.1 ± 5.7	0.12
Smoking, %			<0.01
Current	2	4	
Former	14	12	
Never	84	84	
Type 2 diabetes, %	70.1	43.5	<0.01
Hypertension, %	73.5	48.2	<0.01
Albumin, g/l	53 ± 13	43 ± 6	<0.01
Creatinine, μmol/l	150.1 ± 70.7	70.7 ± 17.6	<0.01
eGFR (MDRD), ml/min/1.73 m^2^	48.0 ± 10.7	107.4 ± 34.5	<0.01
eGFR (CKD-EPI), ml/min/1.73 m^2^	45.5 ± 10.7	103.0 ± 24.5	<0.01
HDL, mmol/l	1.3 ± 0.6	1.2 ± 0.4	<0.01
LDL, mmol/l	4.4 ± 1.6	3.2 ± 1.1	<0.01
Triglyceride, mmol/l	1.7 ± 0.7	1.1 ± 0.7	<0.01

*Values are means ± SD or percentages*.

Using the CKD-EPI equation, the mean (SD) eGFR for those with T2D was 90 (28) ml/min/1.73 m^2^ compared to those without T2D, 105 (28) ml/min/1.73 m^2^, *p*-value <0.001. Based on the CKD-EPI equation, 16.3% of those with T2D and 5.5% of those without T2D had impaired kidney function, *p*-value <0.001. Using the MDRD equation, the mean (SD) eGFR for those with T2D was 95 (37) ml/min/1.73 m^2^ compared to those without T2D, 108 (37) ml/min/1.73 m^2^, *p*-value <0.001. Based on the MDRD equation, 13.4% of those with T2D and 4.8% of those without T2D had impaired kidney function, *p*-value <0.001. The distribution of the characteristics of the study participants by T2D is shown in Table 2. Further analyses were conducted using estimates derived from the MDRD equation.

**Table 2:** **Characteristics of the study participants by type 2 diabetes status**.

Characteristic	Type 2 diabetes status		*p*-value
	Type 2 diabetes	Non-type 2 diabetes	
*n*	2208	2607	
Age, years	55 ± 11	42 ± 15.3	<0.01
Male sex, %	40	42	0.04
Body mass index, kg/m^2^	26.9 ± 5.4	25.5 ± 5.7	<0.01
Smoking, %			<0.01
Current	3	5	
Former	15	9	
Never	82	86	
Hypertension, %	68.3	35.3	<0.01
Albumin, g/l	44 ± 8	44 ± 6	0.37
Creatinine, μmol/l	88.4 ± 44.2	79.6 ± 17.7	<0.01
eGFR (MDRD), ml/min/1.73 m^2^	95.3 ± 36.6	108.1 ± 36.6	<0.01
eGFR (CKD-EPI), ml/min/1.73 m^2^	89.8 ± 27.6	105.1 ± 27.7	<0.01
Impaired kidney function, %	13.4	4.8	<0.01
HDL, mmol/l	1.2 ± 0.5	1.22 ± 0.4	<0.01
LDL, mmol/l	3.5 ± 1.3	3.19 ± 1.2	<0.01
Triglyceride, mmol/l	1.4 ± 0.9	1.1 ± 0.6	<0.01

*Values are means ± SD or percentages*.

In multivariate analyses adjusted for age, sex, BMI, and hypertension, those with T2D had higher risk of impaired kidney function [OR 1.68 (95% CI 1.33–2.12), *p*-value <0.001], compared to those without T2D (Table 3). With further adjustment of the models for lipids (HDLc, LDLc, and triglycerides) in the final model, the risk of impaired kidney function among those with T2D remained significant [OR 1.50 (95% CI 1.17–1.91), *p*-value = 0.001]. The goodness-of-fit test *p*-value was 0.85, suggesting that the final model had a good fit.

**Table 3:** **Association (odds ratios and 95% CI) between type 2 diabetes and impaired kidney function**.

Models	Impaired kidney function, eGFR (MDRD)		*p*-value	Goodness-of-fit *p*-value
	Cases (<60 ml/min/1.73 m^2^)	Controls (≥60 ml/min/1.73 m^2^)		
Age-adjusted model	1.83 (1.45–2.29)	Ref (1.00)	<0.001	0.001
Multivariate model 1	1.70 (1.35–2.15)	Ref (1.00)	<0.001	0.004
Multivariate model 2	1.68 (1.33–2.12)	Ref (1.00)	<0.001	0.06
Multivariate model 3	1.50 (1.17–1.91)	Ref (1.00)	0.001	0.85

*GFR was estimated using the MDRD equation*.

*Logistic regression models were used to estimate the odds ratios and 95% confidence intervals*.

*Goodness of model fit was conducted with Hosmer and Lemeshow goodness-of-fit test*.

*Multivariate model 1 = age, sex, and hypertension*.

*Multivariate model 2 = model 1 + body mass index*.

*Multivariate model 3 = model 2 + lipids (high-, low-density lipoprotein, triglyceride)*.

Next, we conducted stratified analysis to determine any potential effect modification by age, sex, BMI, hypertension, HDLc, LDLc, and triglycerides (Table 4). We observed that participants with T2D had higher risk of impaired kidney function compared to those without T2D, regardless of stratification factors. Furthermore, participants with T2D who were at least 60 years old, female, obese, or hypertensive had higher risk of impaired kidney function compared to those who were not. Participants with T2D had significantly higher risk of impaired kidney function if they had HDLc levels <1.04 mmol/l [OR 1.83 (1.22–2.73), *p*-interaction = 0.005] compared to those without T2D.

**Table 4:** **Association (odds ratios and 95% CI) between type 2 diabetes and impaired kidney function, stratified by key risk factors**.

eGFR	Cases (<60 ml/min/1.73 m^2^)	Controls (≥60 ml/min/1.73 m^2^)	*p* for interaction
Age			
<60 years	1.57 (1.15–2.13)	Ref (1.00)	0.73
≥60 years	2.23 (1.47–3.36)	Ref (1.00)	
Sex			0.88
Male	1.36 (0.92–2.00)	Ref (1.00)	
Female	1.68 (1.23–2.30)	Ref (1.00)	
Body mass index			0.28
Normal	1.51 (1.02–2.22)	Ref (1.00)	
Overweight	1.23 (0.82–1.85)	Ref (1.00)	
Obese	1.93 (1.14–3.27)	Ref (1.00)	
Hypertension			0.17
No	1.23 (0.81–1.86)	Ref (1.00)	
Yes	1.71 (1.26–2.32)	Ref (1.00)	
HDLc			< 0.01
<1.04 mmol/l	1.83 (1.22–2.73)	Ref (1.00)	
≥1.04 mmol/l	1.40 (1.04–1.88)	Ref (1.00)	
LDLc			0.50
<4.14 mmol/l	1.87 (1.35–2.61)	Ref (1.00)	
≥4.14 mmol/l	1.27 (0.89–1.80)	Ref (1.00)	
Triglycerides			0.54
<2.26 mmol/l	1.52 (1.17–1.96)	Ref (1.00)	
≥2.26 mmol/l	2.69 (1.24–5.87)	Ref (1.00)	

*GFR was estimated using the MDRD equation*.

*Logistic regression models were used to estimate the odds ratios and 95% confidence intervals*.

*Each model was adjusted for age, sex, body mass index, hypertension, HDL, LDL, and triglyceride*.

## Discussion

Type 2 diabetes is a known risk factor for CKD; however, there are limited data from SSA on this important relationship. In this study of impaired kidney function in relation to T2D in SSA, about 8.7% of the study participants had impaired kidney function with a higher prevalence in T2D compared to controls (13.4% of those with T2D and 4.8% of those without T2D). Individuals with T2D had 50% significantly increased risk of impaired kidney function after adjustment for covariates, compared to those without T2D. To our knowledge, this is the largest single study of T2D and impaired kidney function in SSA to date.

Although GFR has been established as the most useful overall index of kidney function, proteinuria, an easily measured indicator of glomerular disease, is still used to ascertain acute and CKD in many studies. Many studies from SSA have used proteinuria and/or eGFR measured at one time point as diagnostic criteria for CKD. While measurement of urinary protein has been the most commonly used method in African studies (10), the cutoff for defining kidney impairment has varied between studies, including ≥20 mg/dl (16, 24) or ≥30 mg/dl (8, 12) of protein in urine. A recent systematic review and meta-analysis, including 90 studies from 96 sites in SSA reported that the overall prevalence of CKD in SSA was 13.9% (95% CI 12.2–15.7), the prevalence of CKD varied widely, from as low as 2% in Côte d’Ivoire to as high as 30% in Zimbabwe (10). In the systematic review, several terms were regarded as equivalent to CKD, including renal impairment, renal insufficiency, proteinuria, nephropathy, and end-stage renal disease. The wide variation in the prevalence estimates may be due to differences in study ascertainment criteria, measurement methods, and definitions of CKD. Other implicated causes of CKD in SSA that may explain the geographical variation of CKD prevalence include HIV infection, use of traditional medication or herbal formulations, and chronic glomerulonephritis.

The few studies carried out to examine the burden of kidney impairment defined by GFR among T2D in SSA have been limited by their small sample size and design. A cross-sectional study of 369 participants (94% had T2D) in Mwanza, Tanzania (15) showed that ~25% of the study participants had eGFR <60 ml/min/1.73 m^2^. A review of data from 98 medical records showed that the prevalence of kidney impairment, defined as eGFR <90 ml/min/1.73 m^2^ was 18.1% among T2D in the Democratic Republic of Congo (25). In Nigeria, data from a retrospective sample of 628 patients who were referred to an outpatient clinic with diagnosis of hypertension, diabetes, or both, reported that 38.5% of the patients had eGFR ≤60 ml/min/1.73 m^2^ (14). The present study was larger, including about 5000 participants with prospectively collected data and provided risk estimates using a case–control approach.

Many factors contribute to the risk of impaired kidney function in T2D. In the present study, we observed that individuals with both T2D and hypertension had increased risk of impaired kidney function compared to those with T2D alone. The prevalence of hypertension among T2D participants was 68% in this study. A previous study reported that the prevalence of hypertension among patients with diabetes was as high as 79% (26). Similar to our findings, older age, obesity, and dyslipidemia have been reported as probable risk factors for impaired kidney function in T2D (27). It has been suggested that these risk factors lead to oxidative stress and inflammation, which promote cellular proliferation of endothelial, epithelial, or mesangial cells, resulting in congestion, leukocyte infiltration, glomerular membrane thickening, hyalinization and sclerosis, deposition of fibrin indicating blood leakage, or intra-glomerular thrombosis (28). These changes lead to tissue hypoxia, tissue damage (29) and decline in GFR. In particular, obesity and dyslipidemia may contribute to kidney disease by establishing low-grade inflammation through high expression of lipogenic and pro-inflammatory genes (30). Genetic risk factors also contribute to impaired kidney function. Of particular relevance to our study population is the apolipoprotein L1 (*APOL1*) gene that has been identified as a risk locus for kidney disease in populations of African ancestry, having been first identified in African Americans and subsequently confirmed in studies of sub-Saharan Africans, including Nigerians (31).

This study has several notable strengths, including being the largest single study of impaired kidney function and T2D in SSA thus far, to our knowledge. Importantly, this study provides African risk estimates of impaired kidney function in T2D. Other strengths of the study include adjusting for several lifestyle and biochemical factors known to affect the risk of impaired kidney function, and using standardized tests and validated protocols. Our study has some important limitations. The availability of data on proteinuria on the study participants would have facilitated more fine-grained delineation of kidney damage. Thus, some participants with eGFR above 60 ml/min/1.73 m^2^ and clinical signs of nephropathy may have been misclassified. Also, we did not have data on other implicated causes of kidney impairment in SSA, such as infections, use of traditional medication or herbal formulations and medications, and were unable to assess the impact of these factors on the relationship between T2D and kidney impairment in the sample population. Due to the case–control design of this study, we did not have longitudinal data and also could not rule out the possibility of bias from residual confounding because of unmeasured factors. The associations derived may not infer causality. Large-scale longitudinal studies to determine the incidence, predictors, and genetic risk factors of kidney disease are warranted in African populations, especially among individuals with T2D.

## Conclusion

In conclusion, our study provides evidence for a notable burden of impaired kidney function among T2D in SSA, especially among individuals with dyslipidemia. Given that individuals with T2D often have comorbidities, such as hypertension, obesity, and dyslipidemia, screening for renal impairment should be a priority in the course of their clinical management. Implementation of preventive strategies, early diagnosis and treatment of T2D across SSA populations would facilitate a reduction in the clinical and public health burden of T2D and its comorbidities, including T2D-associated renal impairment.

## Author Contributions

SA conducted the data analyses, interpreted the data, and drafted the manuscript. SA, AAA, AD, FT-A, AB, GC, JZ, DS, OF, GO, BE, KA-B, J Adeleye, WB, AGA, SO, J Acheampong, TJ, JO, CA, and CR designed and implemented the study. AD processed the samples and performed biochemical assays, and JZ collated the data. All the authors contributed to the manuscript, provided critical revisions, approved the final version, and agree to be accountable for all aspects of the work.

## Conflict of Interest Statement

The authors declare that the research was conducted in the absence of any commercial or financial relationships that could be construed as a potential conflict of interest.
